# The *C. elegans* *flr-3(ut9)* mutation is a loss-of-function insertion within the *drl-1* locus

**DOI:** 10.17912/micropub.biology.001047

**Published:** 2023-12-05

**Authors:** Kendra L. Honey, Sarah K. Torzone, Robert H. Dowen

**Affiliations:** 1 Integrative Program for Biological and Genome Sciences, University of North Carolina at Chapel Hill, Chapel Hill, North Carolina, United States; 2 Department of Biology, University of North Carolina at Chapel Hill, Chapel Hill, North Carolina, United States; 3 Department of Cell Biology and Physiology, University of North Carolina at Chapel Hill, Chapel Hill, North Carolina, United States

## Abstract

The genes encoding the mitogen-activated protein kinases DRL-1 and FLR-4 are required for growth and lipid homeostasis in *C. elegans* . Interestingly, the *flr-3 ( ut9 )* mutant, which was previously isolated in a forward genetic screen for mutations that confer fluoride resistance, phenocopies the *drl-1* and *flr-4* loss-of-function mutants; however, the genetic identity of *flr-3* is unknown. Through whole genome sequencing, we found that the *flr-3 ( ut9 )* mutation is an insertion in the *drl-1* locus and disrupts *drl-1* gene function, resulting in dramatic growth defects and impaired vitellogenin production.

**Figure 1. The ut9 allele is an insertion in the drl-1 locus that results in a severe reduction in vitellogenin expression and slowed development:**
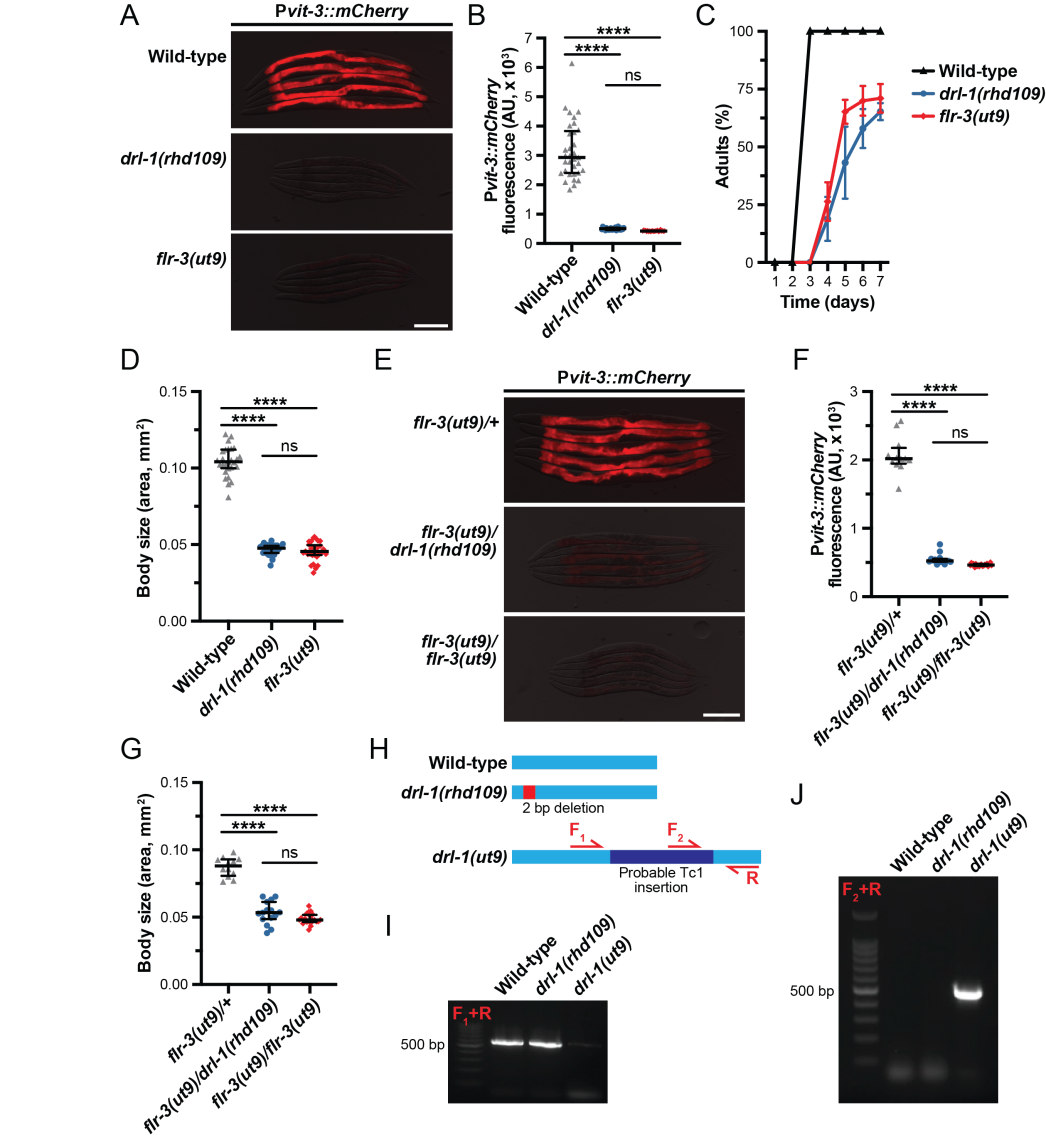
**A)** Representative overlaid DIC and mCherry fluorescence images (scale bar, 200 µm) and **B)** quantification of mCherry fluorescence (median and interquartile range; ****, P<0.0001, one-way ANOVA) of day 1 adult wild-type, *drl-1 ( rhd109 )* , and *flr-3 ( ut9 )* animals expressing the P *vit-3::mCherry* vitellogenesis reporter. **C)** Growth rate (mean +/- SEM, n=3) and **D)** body size (median and interquartile range; ****, P<0.0001, one-way ANOVA) of wild-type, *drl-1 ( rhd109 )* , and *flr-3 ( ut9 )* animals. **E)** Representative overlaid DIC and mCherry fluorescence images (scale bar, 200 µm), **F)** quantification of mCherry fluorescence (median and interquartile range; ****, P<0.0001, one-way ANOVA), and **G)** body size (median and interquartile range; ****, P<0.0001, one-way ANOVA) of F1 cross-progeny derived from P *vit-3::mCherry; flr-3 ( ut9 )* animals crossed to wild-type, *drl-1 ( rhd109 )* , or *flr-3 ( ut9 )* animals. **H)** A schematic showing the genetic lesions within the *drl-1* locus and the primer pairs used to genotype the *ut9* allele. Amplification of the **I)** wild-type or **J)** *ut9* *drl-1* alleles by PCR in wild-type, *drl-1 ( rhd109 )* , and *flr-3 ( ut9 )* animals using the indicated primer pairs. PCR products were resolved by agarose gel electrophoresis. All strains shown here carry the P *vit-3::mCherry* reporter.

## Description

In *C. elegans* , vitellogenesis is the process by which triglycerides and cholesteryl esters are transported from intestinal cells to the germline in the form of lipoprotein particles to support reproduction and embryonic development (Perez and Lehner 2019) . Adult-specific expression of the vitellogenin (VIT) proteins, which is necessary for lipoprotein assembly, is highly regulated and can be tuned by developmental, reproductive, and environmental inputs (DePina et al. 2011; Dowen et al. 2016; Seah et al. 2016; Dowen 2019) .

We previously identified two genes, *drl-1* and *flr-4* , that are required for proper initiation of vitellogenesis at adulthood (Torzone et al. 2023) . Both *drl-1* and *flr-4* encode serine/threonine mitogen-activated protein kinases (MAPK) that are orthologues of mammalian MEKK3 (Take-Uchi et al. 1998; Chamoli et al. 2014) . In addition to vitellogenesis defects, mutation of either gene results in slowed development and a severe reduction in body size (Torzone et al. 2023) . Intriguingly, the *flr-4* mutation was isolated in a forward genetic screen for fluoride resistant mutants along with the *flr-3 ( ut9 )* mutant, which is also slow-growing but is not allelic to *flr-4* (Katsura et al. 1994) .

Given the potential phenotypic similarities between the *flr-3 ( ut9 ),* *drl-1* , and *flr-4* mutants, we sought to identify the unknown *flr-3* locus, as it may encode a novel component of the DRL-1 / FLR-4 MAPK pathway. To assess whether the *flr-3 ( ut9 )* allele phenocopies our previously characterized *drl-1* loss-of-function allele, we first determined vitellogenin expression levels in both mutants using a P *vit-3 ::mCherry* transcriptional reporter. Indeed, we observed a severe reduction in P *vit-3 ::mCherry* expression in both *drl-1 ( rhd109 )* and *flr-3 ( ut9 )* day 1 adult animals ( Figure 1A -B). Additionally, the *drl-1 ( rhd109 )* and *flr-3 ( ut9 )* mutants both displayed a reduced developmental rate and smaller body sizes ( Figure 1C -D). Notably, the vitellogenesis and developmental defects were markedly similar between the *drl-1 ( rhd109 )* and *flr-3 ( ut9 )* mutants.

The striking similarities between *drl-1* and *flr-3* mutant phenotypes suggested that the *flr-3 ( ut9 )* genetic lesion may reside within the *drl-1* locus. To test this hypothesis, we performed a complementation test, crossing *flr-3 ( ut9 )* males carrying the P *vit-3 ::mCherry* reporter to wild-type, *drl-1 ( rhd109 ),* or *flr-3 ( ut9 )* hermaphrodites. The *flr-3 ( ut9 )* allele failed to complement *drl-1 ( rhd109 )* , as the F1 cross progeny displayed dramatically down-regulated P *vit-3 ::mCherry* reporter expression and reduced body sizes ( Figure 1E -G). These phenotypes were as severe as those shown by the *flr-3 ( ut9 )* homozygous mutants. These data indicate that *flr-3 ( ut9 )* is allelic to *drl-1 ( rhd109 )* and that the *ut9* mutation likely disrupts the *drl-1* gene.

To identify the *ut9* mutation, we performed whole genome sequencing of a pool of F2 recombinants after backcrossing *flr-3 ( ut9 )* to wild-type P *vit-3 ::mCherry* animals (Doitsidou et al. 2010) . This approach led to the discovery of an insertion within exon 7 of the *drl-1* coding sequence ( Figure 1H ). Notably, the 5’ and 3’ arms of the insertion match sequences within known Tc1 transposons found throughout the genome, suggesting that the *ut9* allele is likely a Tc1 insertion (see Extended Data for sequences). Because of the repetitive nature and the size of the insertion, we were unable to amplify the full insertion by PCR; and thus, the full sequence of the insertion remains unknown. Using a primer that is positioned within the 3’ end of the insert, we verified that the insertion was specific to the *flr-3 ( ut9 )* strain using PCR (Figure H-J). Moreover, these genotyping results corroborate our whole genome sequencing approach and suggest that the *ut9* mutation is likely a Tc1 insertion within the *drl-1* locus. Thus, we propose renaming the *flr-3 ( ut9 )* mutation to *drl-1 ( ut9 ).*

In conclusion, our results indicate that the *drl-1 ( ut9 )* mutation impairs vitellogenesis, developmental rate, and body size to a similar degree as the *drl-1 ( rhd109 )* frameshift mutation, which we have previously characterized as a strong loss-of-function allele (Torzone et al. 2023) . It is likely that both alleles are null alleles given the size of the *ut9* insertion and where it is positioned in the coding region of the *drl-1* gene. Therefore, while loss of *drl-1* produces severe phenotypes it is unlikely to be an essential gene. This study revealed the identity of the *flr-3 ( ut9 )* mutation and provides a new tool to investigate the DRL-1 / FLR-4 MAPK pathway.

## Methods

*C. elegans strains and maintenance* *C. elegans* strains were cultured at 20ºC on Nematode Growth Media (NGM) plates seeded with *E. coli* OP50 as previously described (Brenner 1974) . The JC53 strain was obtained from the *Caenorhabditis* Genetics Center.

*Fluorescent reporter imaging* L4 animals carrying the P *vit-3 ::mCherry* reporter were picked to fresh *E. coli* OP50 plates and 24 hours later the animals were mounted on a 2% agarose pad with 25mM levamisole. The day 1 adult animals were imaged with a Nikon SMZ-18 Stereo microscope equipped with a DS-Qi2 monochrome camera. The mCherry fluorescence was quantified and analyzed as previously described (Torzone et al. 2023) .

*Growth rate assay* Animals were grown for two generations on NGM plates seeded with *E. coli* OP50 prior to being assayed. Approximately 100 eggs from each strain were picked to the bacterial lawn of a new NGM plate. Animals were scored every 24 hours for 7 days and gravid adults were picked off at each timepoint and recorded as having reached adulthood.

*Body size assay* L4 animals grown for two generations on NGM plates were picked to fresh plates and imaged 24 hours later using a Nikon SMZ-18 Stereo microscope equipped with a DS-Qi2 monochrome camera. Individual animals were traced using the ImageJ2 v2.3.0 software (Rueden et al. 2017) and the two-dimensional outline was used to calculate the body size of each animal in mm ^2^ .

*Complementation test* DLS741 males were crossed to N2 , *drl-1 ( rhd109 )* , or *flr-3 ( ut9 )* hermaphrodites to generate F1 cross-progeny that were heterozygous for the P *vit-3 ::mCherry* reporter. The F1 cross-progeny were picked as L4s and imaged 24 hours later as day 1 adults.

*Whole genome sequencing and genotyping* The *flr-3 ( ut9 )* strain was crossed to DLS537 and F2 recombinants that displayed reduced reporter expression and slow growth were picked and singled to individual NGM plates. Animals from approximately 50 starved F2 plates were pooled (Doitsidou et al. 2010) , the genomic DNA was harvested using the Gentra Puregene Tissue Kit (Qiagen), and the gDNA-Seq libraries were prepared and sequenced using the Illumina NovaSeq platform (Novogene Inc.). The candidate mutations were identified using in-house scripts (Minevich et al. 2012) . Sequencing reads surrounding the insertion site were manually inspected to identify the exact location of the insertion and the sequence of the insertion was manually constructed using overlapping reads. Because the 5’ and 3’ ends of the insertion are identical to other Tc1 transposon sequences found throughout the genome, we were unable to precisely determine the full sequence of the *ut9* insertion from our whole genome sequencing data. Specific amplification of the *ut9* allele was carried out by PCR using the forward primer 5’-GTCATTTCCTTGCAACCTCG-3’ and the universal reverse primer 5’-CCACATCAGCGTGATATCTG-3’, while the wild-type allele was amplified with the forward primer 5’- CCAGAAAATCGACCATCTGC-3’ and the universal reverse primer. The PCR products were resolved by agarose gel electrophoresis and visualized with ethidium bromide.

*Statistics* All statistical analyses (one-way ANOVA with a Bonferroni’s multiple comparisons correction) were performed in Prism v9.

## Reagents

**Table:** 

**Strain**	**Genotype**	**Available From**	**Source**
N2	wild-type	CGC	Brenner 1974
DLS362	*drl-1 ( rhd109 ) IV *	Upon request	Torzone et al. 2023
JC53	*flr-3 ( ut9 ) IV *	CGC	Katsura et al. 1994
DLS537	*rhdSi42 [Pvit-3::mCherry::unc-54 3'UTR + cb-unc-119(+)] II *	Upon request	Torzone et al. 2023
DLS636	*rhdSi42 [Pvit-3::mCherry::unc-54 3'UTR + cb-unc-119(+)] II; drl-1 ( rhd109 ) IV *	Upon request	Torzone et al. 2023
DLS741	*rhdSi42 [Pvit-3::mCherry::unc-54 3’UTR + cb-unc-119(+)] II; flr-3 ( ut9 ) *	Upon request	This study

## Extended Data

Description: A SnapGene file containing annotation of the ut9 allele. Resource Type: Text. DOI: 10.22002/4anmq-cej10

## References

[R1] Brenner S (1974). The genetics of Caenorhabditis elegans.. Genetics.

[R2] Chamoli M, Singh A, Malik Y, Mukhopadhyay A (2014). A novel kinase regulates dietary restriction-mediated longevity in Caenorhabditis elegans.. Aging Cell.

[R3] DePina AS, Iser WB, Park SS, Maudsley S, Wilson MA, Wolkow CA (2011). Regulation of Caenorhabditis elegans vitellogenesis by DAF-2/IIS through separable transcriptional and posttranscriptional mechanisms.. BMC Physiol.

[R4] Doitsidou M, Poole RJ, Sarin S, Bigelow H, Hobert O (2010). C. elegans mutant identification with a one-step whole-genome-sequencing and SNP mapping strategy.. PLoS One.

[R5] Dowen RH (2019). CEH-60/PBX and UNC-62/MEIS Coordinate a Metabolic Switch that Supports Reproduction in C.&nbsp;elegans.. Dev Cell.

[R6] Dowen RH, Breen PC, Tullius T, Conery AL, Ruvkun G (2016). A microRNA program in the C. elegans hypodermis couples to intestinal mTORC2/PQM-1 signaling to modulate fat transport.. Genes Dev.

[R7] Katsura I, Kondo K, Amano T, Ishihara T, Kawakami M (1994). Isolation, characterization and epistasis of fluoride-resistant mutants of Caenorhabditis elegans.. Genetics.

[R8] Minevich G, Park DS, Blankenberg D, Poole RJ, Hobert O (2012). CloudMap: a cloud-based pipeline for analysis of mutant genome sequences.. Genetics.

[R9] Perez MF, Lehner B (2019). Vitellogenins - Yolk Gene Function and Regulation in
*Caenorhabditis elegans*
.. Front Physiol.

[R10] Rueden CT, Schindelin J, Hiner MC, DeZonia BE, Walter AE, Arena ET, Eliceiri KW (2017). ImageJ2: ImageJ for the next generation of scientific image data.. BMC Bioinformatics.

[R11] Seah NE, de Magalhaes Filho CD, Petrashen AP, Henderson HR, Laguer J, Gonzalez J, Dillin A, Hansen M, Lapierre LR (2016). Autophagy-mediated longevity is modulated by lipoprotein biogenesis.. Autophagy.

[R12] Take-Uchi M, Kawakami M, Ishihara T, Amano T, Kondo K, Katsura I (1998). An ion channel of the degenerin/epithelial sodium channel superfamily controls the defecation rhythm in Caenorhabditis elegans.. Proc Natl Acad Sci U S A.

[R13] Torzone SK, Park AY, Breen PC, Cohen NR, Dowen RH (2023). Opposing action of the FLR-2 glycoprotein hormone and DRL-1/FLR-4 MAP kinases balance p38-mediated growth and lipid homeostasis in C. elegans.. PLoS Biol.

